# Measures of trait mindfulness: Convergent validity, shared dimensionality, and linkages to the five-factor model

**DOI:** 10.3389/fpsyg.2014.01164

**Published:** 2014-10-13

**Authors:** A. B. Siegling, K. V. Petrides

**Affiliations:** London Psychometric Laboratory, University College LondonLondon UK

**Keywords:** mindfulness, five-factor model, personality, measures, assessment, psychometrics

## Abstract

This study investigated, and partially aimed to replicate, important construct validity aspects and the homogeneity of trait mindfulness measures. Specifically, the study set out to examine whether a single dimension can explain the shared variance among these measures as well as the extent to which they converge with one another and in terms of their linkages to the five-factor model (FFM). Two samples completed all trait measures of the construct and one of them additionally completed a measure of the Big Five personality traits. Results showed that a single dimension explains the shared variance among measures based on the original, Eastern conceptualization of mindfulness, although not all of them seem to represent this construct comprehensively. Intercorrelations, dimensionality analysis, as well as linkages to the FFM indicated that the Eastern and Western conceptualizations, and their respective measures, reflect distinct constructs. However, the amount of variance overlap with the FFM was similar across the two conceptualizations.

## INTRODUCTION

In its broadest sense, mindfulness can be defined as the extent to which one attends to the present moment, rather than being preoccupied (Brown et al., 2007; Sauer et al., 2012). The concept has received considerable attention in applied and academic psychology, primarily because of its implications for everyday well-being and mental health (e.g., Christopher and Gilbert, 2009; Vujanovic et al., 2010). In applied psychology, it has provided the basis for widespread contemporary approaches to alleviating and treating mental health problems: mindfulness-based cognitive behavior therapy (Segal et al., 2002) and mindfulness-based stress reduction (Kabat-Zinn, 1994). Academic interest in mindfulness has extended to the study of individual differences (i.e., trait mindfulness), accompanied by a recent spurt in psychometric research (Sauer et al., 2012; Bergomi et al., 2013). This research has shown some promising results, with existing measures predicting outcomes such as emotion dysregulation (Vujanovic et al., 2010), sexual body esteem (Fink et al., 2009), insomnia (Ong et al., 2009), nicotine dependence and withdrawal (Vidrine et al., 2009), as well as relationship satisfaction and stress (Barnes et al., 2007).

Scientific research has focused on two conceptualizations of mindfulness originating from different perspectives: (1) the original and traditional conceptualization advanced by Kabat-Zinn (2006), which derives from Eastern philosophy, grounded in Buddhism, and (2) the Western conceptualization of mindfulness developed by Langer (1989). According to Kabat-Zinn (1994, p. 8), mindfulness is “paying attention on purpose, to the present moment, and non-judgmentally.” Langer (1989), on the other hand, has defined mindfulness as “a state in which one is open to novelty, alert to distinctions, sensitive to context, aware of multiple perspectives, and oriented in the present” (Bodner and Langer, 2001, p. 1). A more detailed description and comparison of these two schools of thought was recently presented by Hart et al. (2013).

The set of trait mindfulness measures that has emerged in the literature in recent years can be described as heterogeneous in many respects, indicating that the definition and operationalization of the construct is far from consensual (Sauer et al., 2012; Bergomi et al., 2013; Hart et al., 2013). The present study seeks further to investigate the homogeneity of these scales by examining and, to some extent, cross-validating their convergent validity, shared underlying dimensions, and linkages to the five-factor model (FFM).

### MEASUREMENT OF MINDFULNESS

Extant measures of mindfulness have been described in detail in their respective development studies and recent literature reviews (Sauer et al., 2012; Bergomi et al., 2013; Hart et al., 2013). Importantly, all but one of the scales are based on the original Eastern conceptualization and most of the available measures assess trait mindfulness, including the single measure aligned to the Western conceptualization (Bodner and Langer, 2001; Pirson et al., 2012). In contrast to mindful states that can be actively altered (e.g., by meditation), trait mindfulness refers to a person’s baseline or average mindfulness. Of a total of eight unidimensional scales, one is primarily a state measure requiring prior meditation (Lau et al., 2006), but a trait version has also been developed (Davis et al., 2009).

Beyond the general categorization of mindfulness research based on Eastern versus Western traditions and as state versus trait, a third factor on which existing scales vary (as is the case for most constructs) is on their underlying representation of mindfulness. Even the measures aligned to the Eastern conceptualization are characterized by considerable discrepancy in terms of their underlying models. In the literature of any given construct, the existence and use of multiple measures complicates the comparison and aggregation of research findings, particularly if their underlying models vary substantially. Variations in construct validity, which may be largely unknown, can also lead to inaccurate conclusions when synthesizing findings from different measures.

As discussed elsewhere (Sauer et al., 2012; Bergomi et al., 2013; Hart et al., 2013), certain differences among the existing mindfulness scales in terms of breadth and focus can be found in the mindfulness psychometric literature. Some of the measures are broader in scope, presumably assessing the construct more comprehensively, whereas others have a narrower focus, measuring only some of its elements. Moreover, a few of the measures have subscales, while others provide only a global mindfulness score. Two measures consist of two, more or less, independent subscales, which is problematic, since orthogonal subscales typically represent different constructs (which is why these two measures were not included in the present investigation). Overall, associations among mindfulness scales can be expected to vary considerably, especially for relatively narrow scales assessing certain aspects of the construct. Likewise, correlations with other constructs can be expected to vary across scales, especially if multiple higher-order factors are implicated in the construct. The associations of trait mindfulness measures with higher-order personality factors are particularly informative in regards to understanding their level of similarity and the underlying construct more generally.

### CONVERGENT VALIDITY AND LINKAGES TO THE FFM

Associations among mindfulness scales have been examined in only a few studies. Baer et al. (2006) reported intercorrelations of five mindfulness scales aligned to the Eastern conceptualization, all of which were within a moderate range of 0.31–0.67. As can be expected, the two lowest correlations were reported for a relatively narrow measure, focussing on mindfulness attention and awareness (Brown and Ryan, 2003). Intercorrelations of two particular scales with several others were also within a moderate range (Feldman et al., 2006; Chadwick et al., 2008). The measure based on the distinct Western conceptualization of mindfulness showed weak-to-moderate correlations with two other scales (*r* = 0.27–0.37; Pirson et al., 2012). Generally, evidence for the convergent validity of trait mindfulness scales is restricted to relatively few studies that have examined scale interrelations of only some of the measures, often with the aim of validating a particular scale. It is also unclear whether multiple dimensions explain the observed intercorrelations and, therefore, the shared variance among the scales.

Research into associations between mindfulness and the Big Five personality traits was reviewed in a meta-analysis of 32 samples by Giluk (2009). The focus of that study was exclusively on the Eastern perspective of mindfulness, integrating the results from all measures based on this conceptualization. Of the Big Five, Neuroticism was identified as the strongest correlate of mindfulness (*r* = -0.58), followed by Conscientiousness (*r* = 0.44). Agreeableness also had an average correlation of moderate strength (*r* = 0.30), whereas Extraversion and Openness both correlated weakly with mindfulness (*r* = 0.10 and 0.07, respectively). However, the methodology of that review had several limitations.

One limitation is that the meta-analysis included data from studies that did not report all of the correlations between mindfulness and the Big Five. This practice may have biased the results, with statistical significance leading to the publication of only some of the Big Five’s associations with mindfulness, thus inflating average intercorrelations. Another limitation was the inclusion of a two-dimensional measure comprised of two orthogonal subscales (Cardaciotto et al., 2008). Weakly related factors, let alone unrelated ones, most likely represent multiple dimensions, and using them to represent a single construct has been described as indefensible (Smith et al., 2009). A third possible limitation was the inclusion of subscale correlations with the Big Five, even though composite correlations between multiple subscales and each personality dimension were calculated, presumably to address this problem. Since subscale correlations with the Big Five are likely to vary (between each other and compared to the total mindfulness composite), their inclusion may have led to inaccurate results in regards to global mindfulness. For example, not all mindfulness scales have subscales and it was not stated whether subscale correlations, where examined, were consistently reported for all subscales.

Correlations of the measure aligned to the Western perspective with the FFM were reported in two studies. One of these studies only reported coefficients for Openness and Neuroticism (*r* = 0.73 and -0.27, respectively; Pirson et al., 2012). In the other study, the measure’s correlations with the FFM factors were 0.50 with Openness, -0.21 for Neuroticism, 0.35 for Extraversion, 0.23 for Conscientiousness, and 0.20 for Agreeableness (Bodner and Langer, 2001). This unique pattern of associations with the Big Five, revealing Openness as the strongest correlate, further speaks to the distinctiveness of the measure and the underlying construct. However, more evidence for the measure’s linkages to the FFM, directly in comparison to measures aligned to the Eastern conceptualization of mindfulness, is needed.

In sum, several factors suggest that the relationship between mindfulness and the FFM currently portrayed in the literature may not be fully accurate. First, differences in the construct validity between measures may distort our understanding of the true relationships. Second, to our knowledge, no study has examined the relative “contributions” of relevant higher-order factors, such as the Big Five, to mindfulness. The relative contributions may well differ from the picture created by zero-order correlations, given that the Big Five are not perfectly orthogonal in a statistical sense (e.g., Van der Linden et al., 2012). Last, the file-drawer phenomenon may have influenced the pattern of results reported in Giluk’s (2009) meta-analysis, with non-significant relations (including those of subscales) being under-reported.

### PRESENT STUDY

The present investigation aimed to further examine the homogeneity of existing mindfulness scales and establish whether a single dimension accounts for their shared variance. Two different samples completed all relevant trait measures that yield a global mindfulness score. A related aim was to investigate the linkages of conceptually and dimensionally distinct mindfulness scales to the FFM, addressing some of the limitations of previous research. This aim served to solidify understanding of the level of similarity between existing scales and further elucidate any differences that may exist between underlying dimensions. In contrast to Giluk’s (2009) meta-analysis, only global mindfulness scores were used, which implied the exclusion of two multi-dimensional measures. Taking into account the overlapping variance among the Big Five traits, the unique contributions of the Big Five to mindfulness were examined in addition to bivariate correlations.

## MATERIALS AND METHODS

### PARTICIPANTS AND PROCEDURE

Sample 1 (*N* = 397, 76.0% female) was recruited via the institutional subject pool of a major British university. The mean age was 21.9 years (SD = 5.0), ranging from 18.0 to 57.2 years. Predominantly comprising participants of White – UK heritage or other (53.1%), the sample also included participants from East Asian, (29.6%), South Asian [Indian, Pakistani, and Bangladeshi (8.3%)] backgrounds, as well as a mix of others (8.9%). Participants included undergraduate and Master’s students from various disciplines, though predominantly from psychology and linguistics. Many students received course credit for their participation and, as an additional token of appreciation, were entered into a draw for one of several gift cards. Other students only participated with the incentive of the price draw.

Sample 2 (*N* = 176, 79.5% female) was recruited online using a twofold recruitment procedure in order to obtain a more heterogeneous sample with respect to mindfulness. First, a recruitment notice was posted on participant recruitment platforms for psychological research (e.g., http://www.onlinepsychresearch.co.uk/). Second, two promoters of mindfulness kindly agreed to post a recruitment notice on their twitter pages. The average age of this sample (*M* = 36.37 years, SD = 14.4) was higher than that of Sample 1 and ranged from 15.7 to 76.2 years. Sample 2 was more homogeneous in terms of participant ethnic backgrounds, which were as follows: 84.1% Caucasian, 2.8% East Asian, 1.7% South Asian, 4.5% Black, and 6.8% other/mixed. A price draw of gift cards was offered to participants as a token of appreciation.

Participants of both samples provided demographic information and completed the mindfulness measures described in the next section via an anonymous online survey system. The Sample 1 participants additionally completed the Big Five measure described below. The study was approved by the divisional research ethics board of the authors’ institution.

### MEASURES

All scales were based on self-report, multiple-point response scales, and showed good levels of internal reliability. Internal consistencies for the mindfulness scales are shown in **Table 1**, whereas those for the Big Five are mentioned in the scale description below.

**Table 1:** Internal consistencies and intercorrelations among mindfulness scales in the two samples.

	FFMQ	KIMS	CAMS–R	SMQ	MAAS	FMI	LMS
**Sample 1**
FFMQ	(0.84)					
KIMS	0.90***	(0.80)				
CAMS–R	0.67***	0.60***	(0.74)			
SMQ	0.50***	0.34***	0.52***	(0.80)		
MAAS	0.52***	0.46***	0.44***	0.25***	(0.86)	
FMI	0.59***	0.49***	0.60***	0.49***	0.34***	(0.83)	
LMS	0.33***	0.33***	0.14**	0.00	0.11*	0.21***	(0.82)	
**Sample 2**
FFMQ	(0.92)					
KIMS	0.95***	(0.89)				
CAMS–R	0.77***	0.72***	(0.83)			
SMQ	0.72***	0.66***	0.64***	(0.87)		
MAAS	0.60***	0.59***	0.49***	0.36***	(0.88)	
FMI	0.70***	0.61***	0.75***	0.69***	0.48***	(0.89)	
LMS	0.36***	0.39***	0.25***	0.24**	0.16	0.20*	(0.86)	

*N* = 397 for Sample 1; *N* = 176 for Sample 2, but only 120 participants completed the MAAS and FMI in Sample 2. Coefficient alphas are reported in parentheses along the diagonal for each sample. FFMQ, five facet mindfulness questionnaire (Baer et al., 2006); KIMS, Kentucky Inventory of Mindfulness Skills (Baer et al., 2004); CAMS–R, cognitive and affective mindfulness scale – revised (Feldman et al., 2006); SMQ, Southampton mindfulness questionnaire (Chadwick et al., 2008); MAAS, mindful attention awareness scale (Brown and Ryan, 2003); FMI, freiburg mindfulness inventory (Walach et al., 2006); LMS, langer mindfulness scale (Bodner and Langer, 2001; Pirson et al., 2012).*p < 0.05; **p < 0.01; ***p < 0.001.

#### Five-facet mindfulness questionnaire (FFMQ; Baer et al., 2006)

The FFMQ was developed as a comprehensive measure of the construct, by factor-analysing all of the scales below except the measure based on the Western psychological perspective. This procedure resulted in 39 items distributed across five facets: observing, describing, acting with awareness, non-judging, and non-reactivity. The FFMQ items are rated on a 5-point Likert scale ranging from 1 (*never or very rarely true*) to 5 (*very often or always true*).

#### Kentucky Inventory of Mindfulness Skills (KIMS; Baer et al., 2004)

The KIMS items (also 39) are divided into four facets: observe, describe, act with awareness, and non-reactive stance. All four facets and 24 of the 39 items are now contained within the FMMQ. The KIMS is based on the same 5-point response scale as the FFMQ.

#### Cognitive and affective mindfulness scale – revised (CAMS–R; Feldman et al., 2006)

The CAMS–R global score is also based on four facets (attention, present focus, awareness, and acceptance), each represented by three items (12 in total). The items are rated on a 4-point Likert scale from 1 (*Rarely/Not at all*) to 4 (*Almost Always*).

#### Southampton mindfulness questionnaire (SMQ; Chadwick et al., 2008)

The SMQ consists of 16 items, representing four aspects of mindfulness: mindful observation, letting go of reacting, opening awareness to difficult experience, and acceptance. The response scale of the SMQ ranges from 0 (*Disagree Totally*) to 6 (*Agree Totally*).

#### Mindful attention awareness scale (MAAS; Brown and Ryan, 2003)

The MAAS focuses exclusively on attentional aspects of mindfulness, whereas other scales also incorporate emotional aspects. Fifteen items are responded to on a 6-point Likert scale ranging from 1 (*Almost Always*) to 6 (*Almost Never*).

#### Freiburg mindfulness inventory (FMI; Walach et al., 2006)

The FMI measures mindfulness through 14 items, based on a response scale of 1 (*rarely*) to 4 (*almost always*). The items represent basic aspects of mindfulness: attention to present moment (presence) and non-judgemental attitude (acceptance; Kohls et al., 2009).

#### Langer mindfulness scale (LMS; Bodner and Langer, 2001; Pirson et al., 2012)

A revised 14-item version of the LMS (Pirson et al., 2012), which assesses the Western construct, was used in this study. The items are distributed across three areas (Novelty seeking, engagement, and novelty producing) and responded to on a scale ranging from 1 (*Strongly Disagree*) to 7 (*Strongly Agree*).

#### Big Five inventory (John and Srivastava, 1999)

The Big Five Inventory was selected as a measure of the FFM. Forty-four brief descriptive items are responded to on a 5-point scale, ranging from 1 (*disagree strongly*) to 5 (*agree strongly*). Internal reliabilities were 0.85 for Neuroticism, 0.85 for Extraversion, 0.81 for Openness, 0.71 for Agreeableness, and 0.79 for Conscientiousness.

### STATISTICAL ANALYSIS

After computing intercorrelations among mindfulness scales, we examined if more than a single dimension underlies the shared variance of the mindfulness scales. Excluded from these analyses was the FFMQ, as it derives from the other five scales based on the Eastern conceptualization of the construct. Including the FFMQ in these analyses would duplicate the content of these five measures and bias the results against the LMS. The rest of the scales, including the LMS, were submitted to a principal component analysis.

Bivariate correlations between mindfulness scales and the Big Five as well as average correlations of each Big Five trait with these scales were examined. The LMS was excluded from the average correlations, due to its distinct conceptualization. To assess the unique contributions of the Big Five to mindfulness and the amount of overlap between the FFM and mindfulness, regression analyses were conducted.

## RESULTS

### INTERCORRELATIONS AMONG MINDFULNESS SCALES

Intercorrelations among the mindfulness scales are shown in **Table 1**. These were consistent across the two samples in that for all scales, except the LMS, coefficients exceeded 0.30. The only correlation below this level was between the SMQ and MAAS in Sample 1 (*r* = 0.24). Still, the magnitude of the correlations varied widely: 0.25–0.90 in Sample 1 and 0.36–0.95 in Sample 2. In contrast, correlations between the LMS and the other scales were generally weaker, consistent with the developers’ distinct conceptualization of the construct. Specifically, the LMS had weak average correlations with the other scales in both Sample 1 (*r* = 0.19, range = 0.00–0.33) and Sample 2 (*r* = 0.27, range = 0.16–0.39).

With all six scales shown in **Table 2** included in the principal component analysis, two components emerged in Sample 1 and one component in Sample 2. Due to a high loading of the LMS and negligible loadings from the other scales, the LMS was mainly accountable for the second component in Sample 1 (two of the other scales loaded negatively on this component). Additionally, the LMS had relatively weak loadings on the first component in both samples (λ = 0.34 and 0.32), whereas most of the other scales had loadings of twice this magnitude (λ = 0.63–0.86). These results and the distinct conceptualization of mindfulness underlying the LMS led us to repeat the analysis without the LMS. The results of the reanalysis are shown in parentheses in **Table 2**. Without the LMS, a single principal component accounted for the shared variance among the scales in both samples (56.7 and 67.4%), in each case explaining close to 10% more variance than the analyses with the LMS.

**Table 2:** Principal component analyses of mindfulness scales in the two samples.

Sample	Scale	Factor loading	Communality	(%) of variance
(1)	KIMS CAMS–R SMQ MAAS FMI LMS	0.80 (0.78) 0.85 (0.86) 0.66 (0.68) 0.63 (0.64) 0.79 (0.79) 0.32	0.69 (0.61) 0.74 (0.73) 0.64 (0.67) 0.40 (0.71) 0.62 (0.62) 0.86	48.40 (56.68)

(2)	KIMS CAMS–R SMQ MAAS FMI LMS	0.86 (0.85) 0.86 (0.87) 0.79 (0.80) 0.69 (0.69) 0.86 (0.87) 0.34	0.74 (0.73) 0.75 (0.76) 0.63 (0.64) 0.47 (0.48) 0.75 (0.76) 0.12	57.60 (67.42)

**N* = 397 for Sample 1 and 120 for Sample 2. Results shown in parentheses derive from analyses excluding the LMS, which loaded highly on a second component in oxSample 1 on which it loaded highly (λ = 0.87) and relatively weakly on the first component in both samples. KIMS, Kentucky Inventory of Mindfulness Skills (Baer et al., 2004); CAMS–R, cognitive and affective mindfulness scale – revised (Feldman et al., 2006); SMQ, Southampton mindfulness questionnaire (Chadwick et al., 2008); MAAS, mindful attention awareness scale (Brown and Ryan, 2003); FMI, freiburg mindfulness inventory (Walach et al., 2006); LMS, langer mindfulness scale (Bodner and Langer, 2001; Pirson et al., 2012).*

### MINDFULNESS AND THE BIG FIVE

Bivariate correlations between mindfulness scales and the Big Five are shown in **Table 3**. Extraversion and Conscientiousness correlated with all of the mindfulness scales. Neuroticism correlated with all of the scales based on the Eastern conceptualization, but not with the LMS. Agreeableness correlated with all scales except for the SMQ. Openness was the least reliable correlate of the mindfulness scales based on the Eastern conceptualization; it correlated with the FFMQ, KIMS, FMI, but not with the CAMS–R, SMQ, and MAAS. In contrast, it was the strongest personality correlate of the LMS. All significant correlations were in an expected direction. Neuroticism was the only Big Five dimension showing moderately strong correlations with all mindfulness scales based on the Eastern conceptualization (*r* = -0.32 – -0.58). The other four dimensions showed a mix of weak-to-moderate correlations (*r* = 0.12–0.42). The LMS’ correlation with Openness was the strongest in the matrix (*r* = 0.67). However, its other significant correlations with personality dimensions were all relatively weak (*r* = 0.15–0.24).

**Table 3:** Bivariate correlations between mindfulness scales and the Big Five in Sample 1.

	FFMQ	KIMS	CAMS–R	SMQ	MAAS	FMI	LMS
Neuroticism Extraversion Openness Agreeableness Conscientiousness	-0.47*** 0.34*** 0.31*** 0.27*** 0.37***	-0.32*** 0.32*** 0.35*** 0.26*** 0.36***	-0.52*** 0.15** 0.05 0.21*** 0.42***	-0.58*** 0.16** -0.01 0.08 0.12*	-0.35*** 0.14** 0.02 0.31*** 0.31***	-0.55*** 0.24*** 0.21*** 0.22*** 0.16**	-0.08 0.24*** 0.67*** 0.15** 0.19***

*N* = 358. FFMQ, five facet mindfulness questionnaire (Baer et al., 2006); KIMS, Kentucky Inventory of Mindfulness Skills (Baer et al., 2004); CAMS–R, cognitive and affective mindfulness scale – revised (Feldman et al., 2006); SMQ, Southampton mindfulness questionnaire (Chadwick et al., 2008); MAAS, mindful attention awareness scale (Brown and Ryan, 2003); FMI, freiburg mindfulness inventory (Walach et al., 2006); LMS, langer mindfulness scale (Bodner and Langer, 2001; Pirson et al., 2012). *p < 0.05; **p < 0.01; ***p < 0.001.

Average correlations of the Big Five with the mindfulness scales, excluding the LMS, were as follows: -0.46 for Neuroticism, 0.22 for Extraversion, 0.15 for Openness, 0.22 for Agreeableness, and 0.29 for Conscientiousness.

The inconsistent magnitude of associations among the mindfulness scales reflects previous findings and suggests that not all scales are measuring mindfulness to the same degree. Consequently, linkages of mindfulness to the FFM were not separately examined for all scales, since differences in the breadth of these measures could lead to divergent patterns of associations and uncertainty about the relationships between mindfulness and the FFM. Since all scales loaded on a single component, a composite of the KIMS, CAMS–R, SMQ, MAAS, and FMI was derived from the principal component analysis described above, excluding the LMS. The FFMQ was examined separately as a way of cross-validation; it derives from these five scales and showed good convergence with their composite at 0.85 in Sample 1 and 0.90 in Sample 2. The LMS’ linkages to the Big Five were also examined in a separate analysis due to the distinct conceptualization of mindfulness underlying this scale.

The regression analysis results are summarized in **Table 4**. Beta weights for the Big Five were consistent in order of magnitude between the FFMQ and the multi-scale composite. Specifically, the order of predictors in terms of strength was Neuroticism, Conscientiousness, Openness, Extraversion, and Agreeableness. Extraversion was a significant predictor of the FFMQ only and Agreeableness did not show a significant effect on either variable. The remaining personality dimensions reached significance in both analyses. Overall, personality explained 43 and 51% of the mindfulness variance in the FFMQ and multi-scale composite scores, both of which represent the Eastern conceptualization of mindfulness.

**Table 4:** Regressions of the FFMQ, multi-scale composite, and LMS on the Big Five in Sample 1.

	FFMQ		MSC		LMS	
	*F*(5,352) = 54.04**		*F*(5,352) = 72.11**		*F*(5,352) = 68.22**	
Predictor	β	*R*^2^	β	*R*^2^	β	*R*^2^
Neuroticism Extraversion Openness Agreeableness Conscientiousness	-0.38** 0.17** 0.28** 0.05 0.28**	0.43	-0.56** 0.07 0.15** 0.05 0.27**	0.51	-0.04 0.11* 0.65** 0.02 0.16**	0.49

*N* = 358. Regression coefficients (β) represent standardized beta weights. FFMQ, five facet mindfulness questionnaire (Baer et al., 2006); MSC, multi-scale composite; LMS, langer mindfulness scale (Bodner and Langer, 2001; Pirson et al., 2012). *p < 0.01; **p < 0.001.

While personality explained a similar amount of variance in the LMS (49%), which is aligned to the Western model, a very different pattern of predictive effects was observed. In this case, Openness was by far the strongest predictor, followed by Conscientiousness and Extraversion. The beta weights for Neuroticism and Agreeableness were not significant.

## DISCUSSION

The present study aimed to clarify issues surrounding the conceptualization and measurement of trait mindfulness, particularly the similarity of the extant measures. The first issue concerned the measures’ convergent validity. Although correlations among measures aligned to the Eastern perspective were generally within a moderate-to-strong range, there were considerable discrepancies. These results are consistent with previous findings (Baer et al., 2006; Feldman et al., 2006; Chadwick et al., 2008) and suggest that some measures represent the construct partially, while others represent it more comprehensively. Intercorrelations involving the LMS were noticeably lower than those of the other scales, as could be expected given its distinct conceptualization of mindfulness and previous findings (Pirson et al., 2012). These results indicate that the LMS shares the least amount of variance with the other measures.

The second issue concerned whether a single dimension can account for the shared variance between mindfulness scales. The results from both samples showed that the shared variance of the scales based on the original, Eastern conceptualization of mindfulness is explained by a single dimension, which presumably represents the target construct. In contrast, and consistent with the bivariate correlations across the two samples, the LMS loaded relatively weakly on this factor and even produced a second factor in Sample 1, on which it loaded highly. These results strongly suggest that the two conceptualizations of mindfulness represent distinct constructs.

The third issue concerned the pattern of relationships between the various measures of mindfulness and the Big Five personality dimensions. Previous research has been mostly restricted to the Eastern conceptualization, with a heterogeneous set of scales imposing some limitations to the interpretability of findings. Results were consistent with Giluk’s (2009) meta-analysis in that Neuroticism showed the strongest, and Conscientiousness the second strongest, relationship with the multi-scale composite and FFMQ total scores. In contrast to Giluk’s (2009) results, however, which revealed Extraversion as the weakest correlate, the weakest average correlate in our sample was Openness; Extraversion showed the same magnitude of association as Agreeableness, which was the third strongest correlate in Giluk’s (2009) meta-analysis. These differences may have several explanations. First and foremost, our results involving the Big Five are based on a single sample and on a single measure of the Big Five traits, whereas Giluk integrated the results of multiple samples spanning various Big Five measures. On the other hand, as mentioned in the introduction, Giluk’s (2009) meta-analysis had certain limitations, including possible file-drawer effects and the inclusion of a mindfulness scale comprised of orthogonal factors.

An advantage of the present investigation is that it examined the unique contributions of the Big Five to trait mindfulness. Since the five unidimensional scales based on the Eastern conceptualization of mindfulness loaded on a single component, a multi-scale composite (rather than each constituent scale) was used in the present study to examine the linkages of the underlying dimension to the Big Five. The strategic benefit of this approach was that this composite should yield a more comprehensive representation of the construct and reveal its linkages to the FFM more accurately than individual measures. In addition, the FFMQ was examined separately, because it was empirically derived from these scales (Baer et al., 2006) and, thus, useful for cross-validation purposes.

When regressing the two very similar variables representative of the Eastern conceptualization (the FFMQ global score and the composite derived from the other unidimensional scales) on the Big Five, a slightly different picture emerged compared to the zero-order correlations. While Neuroticism and Conscientiousness remained the strongest predictors, Openness, which showed the weakest average correlation, became the third strongest predictor of the multi-scale composite and, together with Conscientiousness, the second strongest predictor of the FFMQ global score. Surprisingly, Agreeableness had no significant predictive effects on either variable. Extraversion predicted the FFMQ, but not the multi-scale composite. These variables shared about half their variance with the FFM.

The LMS’s pattern of associations with the FFM was very different from that observed for the FFMQ and multi-scale composite scores. Neuroticism was the weakest and sole non-significant correlate, despite previous reports of small, but significant, correlations with the LMS (Bodner and Langer, 2001; Pirson et al., 2012). Openness (the weakest average correlate of the other variables) was by far the strongest correlate of the LMS. The strong association with Openness is not surprising, given the nature of the model and its subscales (novelty producing, novelty seeking, and engagement), which reflect this basic personality dimension. Also, similar associations with Openness were previously reported in Pirson et al. ( 2012; *r* = 0.73) and Bodner and Langer (2001; *r* = 50). The remaining Big Five traits had significant, but relatively weak, correlations with the LMS, again of similar magnitude as correlations reported previously (Pirson et al., 2012). Regression analysis suggested a similar conclusion, except that Agreeableness did not predict unique LMS variance with the other four personality factors in the regression equation. These results suggest that Agreeableness is not incrementally related to mindfulness.

The findings speak to the distinctiveness of the LMS from the other scales, consistent with the scale developers’ divergent conceptualization of mindfulness. Although the results indicate some overlap of the LMS with the other scales, as has been previously found (Pirson et al., 2012), it appears that most of the variance in its global composite score is due to a different dimension. In terms of linkages to the Big Five, unique to the LMS is its strong correlation with Openness. In view of these and previous relevant results, it seems prudent for future research to explicitly and systematically differentiate between the Eastern and Western conceptualizations of mindfulness.

A single dimension explains the shared variance of the scales aligned to the original, Eastern conceptualization of mindfulness and factor loadings suggest that they all tap into the same construct, albeit to different extents. Some of the scales seem to assess different parts of the construct, notably the SMQ and MAAS, which had relatively weak correlations with the other scales. For comprehensive measurement of the Eastern conceptualization of mindfulness, the FFMQ, KIMS, and CAMS-R seem to be the best options at present, whereas the MAAS appears to be least comprehensive, consistent with its relatively narrow focus on mindful attention and awareness.

Some limitations of the present study must be noted, particularly in regards to the examination of linkages between mindfulness and the FFM. Unlike previous studies, our conclusions regarding these linkages are based on a single sample that was also relatively homogenous. A second limitation is the exclusive reliance on a single measure of the FFM. It is possible that the Big Five Inventory used in our study may not represent the Big Five as accurately or comprehensively as other measures used in previous studies. An updated meta-analysis addressing the limitations of Giluk’s (2009) study would shed light on the validity of our results pertaining to measures aligned to the Eastern conceptualization of mindfulness.

Despite limitations in existing research on personality and mindfulness, some conclusions can be drawn with relatively high confidence from the consistent findings. First, both Giluk’s (2009) study and ours suggest that Neuroticism, followed by Conscientiousness, are the two strongest personality correlates of mindfulness, as conceptualized in the Eastern perspective. Second, although the shared variance between the FFM and mindfulness was not assessed in Giluk’s study, the magnitude of associations reported in her study are similar to ours. Trait mindfulness, thus, seems to share considerable variance with the FFM, which our results indicate to be around 50%. Third, linkages of the Big Five to the mindfulness construct based on Langer (1989) Western conceptualization appear to be different from those of the Eastern conceptualization advanced by Kabat-Zinn’s (1994); Openness is the predominant personality factor in this construct. Last, the shared variance of measures based on the original perspective seems to be reflecting a single dimension that is largely unrelated to the LMS. Collectively, these findings speak to the distinctiveness of the two mindfulness conceptualizations and their respective measures.

## Conflict of Interest Statement

The authors declare that the research was conducted in the absence of any commercial or financial relationships that could be construed as a potential conflict of interest.
